# Network Pharmacology and Molecular Docking Analysis of the Mechanism Underlying Yikunyin's Therapeutic Effect on Menopausal Syndrome

**DOI:** 10.1155/2022/7302419

**Published:** 2022-06-06

**Authors:** Xin Tan, Yan-Ping Du, Qian Luo, Xue-Bing Zhan, Yun-Shu Kuang, Xiao Liang, Yun Zhang, Lin Wang, Bing Chen, Ming Wen

**Affiliations:** ^1^Department of Pathology, Wannan Medical College, Wuhu 241002, Anhui Province, China; ^2^Department of Gynaecology and Obstetrics, The First Peoples Hospital of Wuhu City, Wuhu 241001, Anhui Province, China

## Abstract

**Objective:**

Yikunyin is an empirical prescription that exhibits good efficacy in the clinical treatment of menopausal syndrome; however, its underlying mechanism remains unclear. This study investigates the mechanism implicated in the therapeutic effect of Yikunyin by identifying its hub genes, central pathways, and key active ingredients.

**Method:**

The active ingredients and targets of Yikunyin were obtained from the Traditional Chinese Medicine Systems Pharmacology database, whereas the targets related to menopausal syndrome were obtained from GeneCards, PharmGKB, Therapeutic Target Database (TTD), and Comparative Toxicogenomics Database (CTD). To reveal the pharmacological mechanism, the component-target and the intersecting protein-protein interaction (PPI) networks were constructed, and Gene Ontology (GO) and Kyoto Encyclopedia of Genes and Genomes (KEGG) analyses were performed. Finally, molecular docking was carried out to assess the strength of binding between the key active ingredients and key targets.

**Results:**

A total of 418 targets and 121 active ingredients were identified in Yikunyin. The intersection of Yikunyin's 418 targets with the 2822 targets related to menopausal syndrome shows that there are 247 common targets that can be considered potential targets of Yikunyin in the treatment of menopausal syndrome. The topology analysis of the constructed PPI network conducted using the Cytoscape software shows that there are 15 hub genes implicated in the therapeutic effect of Yikunyin: AKT1, PRKCA, TLR9, CXCL10, PRKCD, PARP1, ABCB1, TP53, CAV1, MAPK8, PPARA, GRB2, EGFR, IL-6, and JAK2. Moreover, the key active components acting on these genes are paeoniflorin, luteolin, quercetin, beta-sitosterol, and kaempferol. GO and KEGG analyses indicate that Yikunyin can treat menopausal syndrome by regulating cellular response to chemical stress (GO:0062197), cellular response to oxidative stress (GO:0034599), phosphatase binding (GO:0019902), cytokine receptor binding (GO:0005126), PI3K-Akt signaling (hsa04151), lipid and atherosclerosis (hsa05417), and hepatitis B (hsa05161). Finally, the results of molecular docking suggest that the key active ingredients and key targets can bind well, with binding energies of less than −5 kJ/mol.

**Conclusion:**

The research conducted herein reveals that Yikunyin treats menopausal syndrome by targeting AKT1 and IL-6 and by regulating the PI3K-Akt signaling pathway. Moreover, it provides a new idea for understanding the therapeutic effects of traditional Chinese medicines.

## 1. Introduction

Menopause is a physiological stage characterized by the permanent cessation of a woman's menstrual period due to the loss of ovarian follicle function. According to a global project comprising 36 international studies conducted in 24 countries across 6 continents, the mean age of natural menopause onset ranges between 46 and 52 years, with an overall average of 48.8 years [1]. During the menopausal transition, women experience a variety of physical, psychological, and social symptoms, such as depression, insomnia, osteoarthritis, and atherosclerosis. These symptoms occur frequently and are usually associated with a worsening quality of life, both at home and at work [2–4]. As one of the most important therapies in the field of complementary and alternative medicine, traditional Chinese medicine (TCM) makes use of naturally occurring, plant-based ingredients that are not industrially processed. Consequently, it has become increasingly popular in the treatment of diseases, including menopausal and perimenopausal syndrome, in China and around the world [5]. In Western countries, hormone therapy is most commonly used to treat menopausal symptoms, with an incidence of 19.5%. However, this therapy can cause many adverse reactions, such as increased risk of breast cancer, dyslipidemia, and osteoporosis, and thus, it must be used with great caution [6]. Considering that the side effects of TCM are few and that its efficiency is high, this natural medicine constitutes a feasible candidate for the treatment of menopausal syndrome.

Yikunyin is a Gui Shen Pill-based formula composed of nine herbs, namely Gou Qi Zi (Lycii Fructus), Fu Ling (Poria), Bai Shao (Paeoniae Radix Alba), Xian Ling Pi (Epimedii Folium), Huang Qi (Astragali Radix), Shu Di Huang (Rehmanniae Radix Praeparata), Gou Teng (Ramulus Uncariae Cum Uncis), Muli (oysters), and He Huan Pi (Albizia Peel). These herbs are rich in flavonoids, which are known for their anti-inflammatory, antioxidant, anticancer, and antidiabetic effects [7, 8]. Clinical studies have shown that Yikunyin can significantly alleviate the symptoms of menopausal syndrome in patients, with a total effective rate of 81.25% after treatment [9]. However, the underlying mechanism remains unclear. Therefore, to promote the clinical application of Yikunyin, its therapeutic mechanism must be elucidated.

Unlike Western medicines, TCM prescriptions, such as Yikunyin, comprise a variety of herbs. Considering their complex composition, the effects of these prescriptions cannot be readily studied using simple methods. In 2008, Hopkins proposed network pharmacology as an effective method that can be used to study the molecular mechanisms of complex systems (e. g., natural herbs and traditional Chinese medicines) by establishing the relationships between multiple compounds and targets [10]. In this study, we explore the effect of Yikunyin in treating menopausal syndrome, and we systematically evaluate its therapeutic targets and mechanisms using network pharmacology and molecular docking.

## 2. Materials and Methods

### 2.1. Screening for the Active Chemical Constituents in Yikunyin

The chemical composition of Yikunyin was obtained from the Traditional Chinese Medicine Systems Pharmacology (TCMSP) database (https://tcmspw.com/tcmsp.php) [11], the Shanghai Institute of Organic Chemistry of Chinese Academy of Science, the Chemistry (CASC) database (https://202.127.145.134/scdb/), and a high-throughput experiment- and reference-guided database of traditional Chinese medicine (HERB) database (http://herb.ac.cn/). The oral bioavailability (OB) and drug-likeness (DL) of each component were obtained from the TCMSP database. Since Yikunyin is administered orally, OB, a measure of the drug's ability to enter systemic circulation, is an important indicator [12]. Drug-likeness is also important, as it helps reduce the cost of drug discovery. Of the numerous tangible small molecules in herbs, only those that meet the drug-likeness criterion are considered for further research [13]. In this study, the criteria of OB ≥ 30% and DL ≥ 0.18 were used to screen the active ingredients in Yikunyin [14].

The Swiss Absorption, Distribution, Metabolism, and Elimination (ADME) system (https://www.swissadme.ch/) provides pharmacokinetic and drug-likeness data of compounds not included in the TCMSP database. In this research, Swiss ADME was used to identify the active compounds in Yikunyin based on GI absorption and drug-likeness. In particular, the compounds with “High” GI absorption and more than three “Yes” drug-likeness criteria were considered to be active.

### 2.2. Determining the Targets of Yikunyin in the Treatment of Menopausal Syndrome

The TCMSP database and SwissTargetPrediction (https://www.swisstargetprediction.ch/) were used to predict the relevant targets of Yikunyin. The compound SDF files required for SwissTargetPrediction were obtained from PubChem (https://pubchem.ncbi.nlm.nih.gov/). The UniPort database (https://www.uniprot.org/) was used to convert protein targets into genetic targets, species restricted to humans, and reviewed targets.

As for the targets of menopausal syndrome, they were collected from four databases, namely GeneCards (https://www.genecards.org/), PharmGKB (https://www.pharmgkb.org/), the Therapeutic Target Database(TTD) (https://db.idrblab.net/ttd/), and Comparative Toxicogenomics Database(CTD) (https://ctdbase.org/), using the keyword “menopausal syndrome.” The identified menopausal syndrome targets were intersected with the drug targets using the online website https://bioinformatics.psb.ugent.be/webtools/Venn/. The resulting Venn diagram was visually processed, and the intersection targets were considered to be the targets of Yikunyin in the treatment of menopausal syndrome.

### 2.3. Construction and Analysis of the Network

#### 2.3.1. PPI Network

To construct the intersecting protein-protein interaction (PPI) network, the identified targets were entered into the STRING database (https://string-db.org). The organism criterion was set to “Homo sapiens,” and the minimum required interaction score was fixed at 0.7. For visualization and topology analysis, the resulting TSV files were downloaded from the STRING database and uploaded into Cytoscape 3.7.2. The important targets in the network were identified based on their degree value (large degree) [15].

#### 2.3.2. Identification of Hub Genes

CytoHubba is a Cytoscape plug-in that uses 12 algorithms (betweenness, bottleneck, closeness, clustering coefficient, degree, DMNC, eccentricity, EPC, MCC, MNC, radiality, and stress) to characterize the nodes in the PPI network and to identify the central elements based on their importance [16]. After constructing the PPI network of common targets, the 12 algorithms in CytoHubba were used for topology analysis. The top-ranking targets under each algorithm were selected as hub genes, and the active compounds corresponding to these genes were identified as the key active compounds in Yikunyin [17].

#### 2.3.3. GO and KEGG Pathway Enrichment Analyses

Gene Ontology (GO) analysis was used to identify the genes implicated in biological process (BP), cell composition (CC), and molecular function (MF) related to menopausal syndrome, whereas the Kyoto Encyclopedia of Genes and Genomes (KEGG) analysis was used to identify the corresponding pathways. The analyses were carried out using the clusterProfiler *R* package. *P* values below 0.01 were considered statistically significant [18].

#### 2.3.4. Verification by Molecular Docking

To predict whether the key active compounds can bind well to the key protein targets, molecular docking analysis was carried out using AutoDockTools 1.5.6. The molecular structures of the key active compounds were taken from PubChem (https://pubchem.ncbi.nlm.nih.gov/), whereas the structures of proteins were obtained from the Protein Data Bank (PDB, https://www.rcsb.org/). The PDB files of proteins and drugs were uploaded into AutoDockTools 1.5.6 for pretreatment (e.g., deleting water and hydrogenation), and then, molecular docking was conducted to determine the binding energy. The obtained PDBQT files were converted to PDB files and imported into PyMOL 2.2.0 for visual processing.

## 3. Results

### 3.1. Screening for the Active Chemical Constituents in Yikunyin

Based on ADME screening and searches conducted in TCMSP, CASC, and HERB databases, the nine herbs in Yikunyin comprise 121 active components (common active ingredients in two or more Chinese medicines are counted only once), eight of which are in Bai Shao, six in Fu Ling, 32 in Gou Teng, 35 in Gou Qi Zi, 16 in Huang Qi, two in Shu Di, 21 in Xian Ling Pi, 10 in He Huang Pi, and seven in Muli. These components are listed in Supplementary Table 1.

### 3.2. Determining the Targets of Yikunyin in the Treatment of Menopausal Syndrome

**Figure 1:**
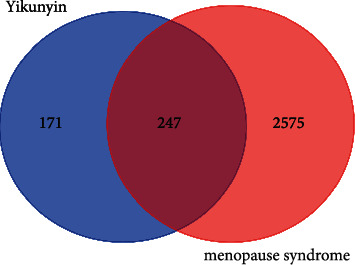
Diagram showing the 247 common targets among the 418 targets of Yikunyin and 2822 targets of menopausal syndrome.

In total, 418 targets corresponding to the 121 active ingredients in Yikunyin were identified using the TCMSP database and SwissTargetPrediction. Based on searches conducted in the GeneCards, CTD, PharmGKB, and TTD databases, a total of 2,822 targets are associated with menopausal syndrome. Of these targets, 247 are also associated with Yikunyin, as shown in the Venn diagram presented in Figure 1.

### 3.3. Common Target-Component Network Construction

**Figure 2:**
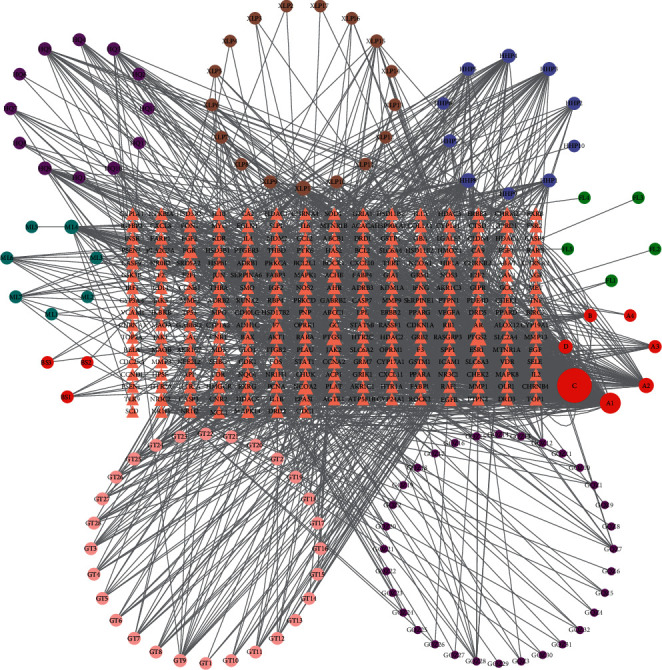
Common target-active ingredient network. The circles and triangles represent the active ingredients in Yikunyin and the common targets, respectively, and the lines indicate relationships between nodes. Different colors are used to pinpoint the active ingredients in different herbs. The molecular IDs corresponding to signs in circles are listed in Supplementary File 2. Nodes A1, A2, A3, and A4, (B), (C), and (D) represent the common components of two or more herbs.

To better understand the relationship between TCM components and targets, a common target-component network was constructed, as shown in Figure 2. The circular and triangular nodes in this network represent the active ingredients of Yikunyin and the common targets, respectively, whereas the lines signify relationships between nodes. Different colors are used for ingredients belonging to different herbs. The molecular IDs corresponding to signs in circular nodes are given in Supplementary Table 2. In total, the network comprises 368 nodes, including 247 nodes of common targets and 121 nodes of active ingredients. Using the CytoHubba plug-in in Cytoscape, the degree value of each node in the network was analyzed and ranked. The top five active ingredients were found to be quercetin (degree = 420), kaempferol (degree = 160), beta-sitosterol (degree = 51), luteolin (degree = 44), and norarmepavine (degree = 43).

### 3.4. In-Depth Analysis of Common Targets

#### 3.4.1. PPI Network of Common Targets and Identification of Hub Genes

**Figure 3:**
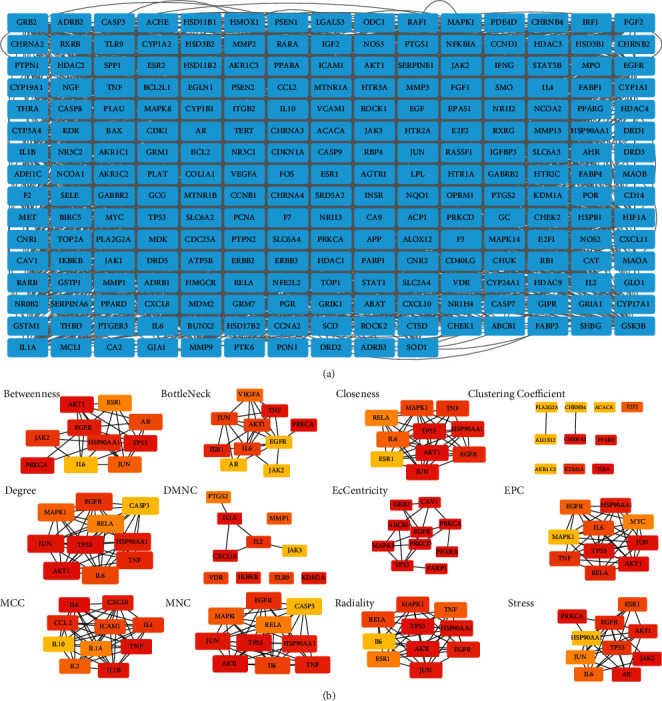
“(a) PPI network of the 247 common targets. (b) Topology analysis of the 12 subnetworks generated using the 12 algorithms in CytoHubba, a Cytoscape plug-in. Deeper red colors signify higher algorithm scores and more important targets”.

**Figure 4:**
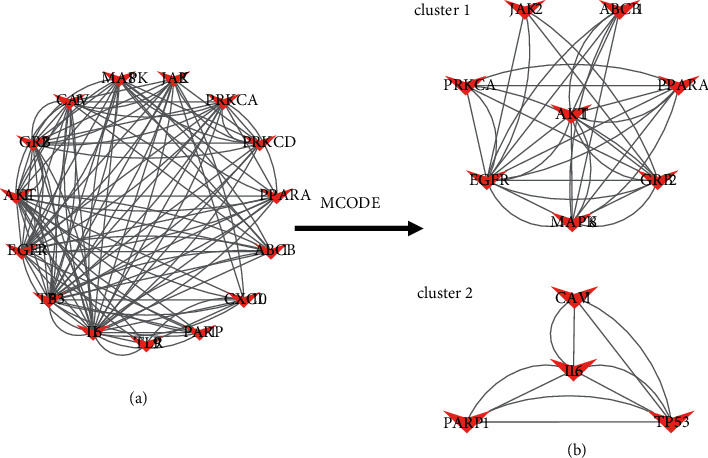
“(a) Hub gene PPI network. (b) The two modules of the hub gene PPI network determined using the MCODE plug-in. AKT1 and IL-6 in the center are seed nodes”.

The PPI network was constructed by inputting all 247 common targets into the STRING database, and it was visualized using Cytoscape (Figure 3(a)). Topology analysis of the constructed network was carried out using the CytoHubba plug-in, and 12 subnetworks were obtained using 12 algorithms (betweenness, bottleneck, closeness, clustering coefficient, degree, density of maximum neighborhood component (DMNC), eccentricity, edge percolated component (EPC), maximal clique centrality (MCC), maximal clique centrality (MNC), radiality, and stress). Each subnetwork contains the top ten targets under the corresponding algorithm, as shown in Figure 3(b) (deeper red color signifies higher score). The ranking of targets under different algorithms is provided in Supplementary File 3. The top-ranking targets in the 12 subnetworks (in eccentricity, 10 genes rank first, so we take these 10 genes into consideration, that is why there are 15 top-ranking genes in 12 subnetworks) were considered hub genes that play an important role in Yikunyin treatment of menopausal syndrome. These genes are AKT1, PRKCA, TLR9, CXCL10, PRKCD, PARP1, ABCB1, TP53, CAV1, MAPK8, PPARA, GRB2, EGFR, IL-6, and JAK2 (Figure 4(a)). Figure 4(b) shows the two functional modules obtained by analyzing the hub genes using the MCODE plug-in. Clearly, AKT1 and IL-6 are the seeds of the two functional modules, which suggest that these two targets may play a pivotal role in Yikunyin's therapeutic effect.

#### 3.4.2. Screening for Key Active Compounds and Construction of the Hub Gene-Component Network

**Figure 5:**
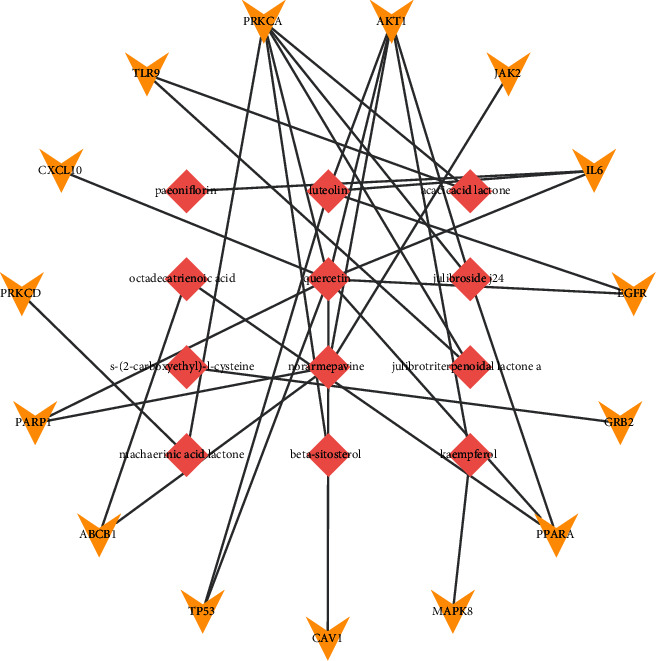
“Hub gene-component network. The yellow and pink nodes represent the 15 hub genes and the active ingredients acting on them, respectively”.

The key compounds corresponding to the 15 hub genes identified herein were determined by a reverse search, and they are paeoniflorin, luteolin, octadecatrienoic acid, quercetin, beta-sitosterol, kaempferol, acacic acid lactone, julibroside j24, julibrotriterpenoidal lactone A, norarmepavine, S-(2-carboxyethyl)-L-cysteine, and machaerinic acid lactone.  Figure 5 presents the hub gene-component network consisting of 27 nodes (15 hub genes and 12 key compounds).

#### 3.4.3. GO and KEGG Pathway Enrichment Analyses of Hub Genes

**Figure 6:**
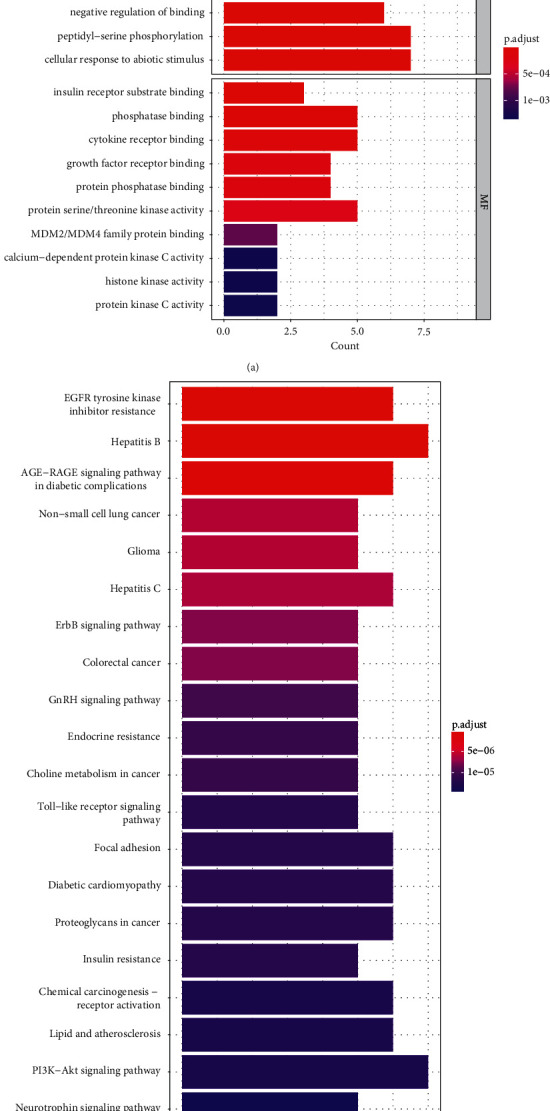
“(a) GO and (b) KEGG enrichment analyses of hub genes. The deeper the red color, the smaller the *P* value, the longer the bar, and the greater the enrichment of the gene”.

Since the 15 hub genes play important roles in the treatment of menopausal syndrome by Yikunyin, it is necessary to identify the pathways in which these genes are enriched. Based on GO and KEGG analyses, the hub genes are implicated in 644 GOs (624 BPs and 20 MFs) and 91 KEGGs.  Figure 6 shows the top 10 most statistically significant BPs and MFs, as well as the top 20 most statistically significant KEGG pathways (sorted based on *P* value). The obtained results indicate that the mechanism of menopausal syndrome treatment by Yikunyin is mainly related to cellular response to chemical stress (GO:0062197), cellular response to oxidative stress (GO:0034599), phosphatase binding (GO:0019902), cytokine receptor binding (GO:0005126), PI3K-Akt signaling (hsa04151), lipid and atherosclerosis (hsa05417), and hepatitis B (hsa05161).

#### 3.4.4. Molecular Docking Validation

**Figure 7:**
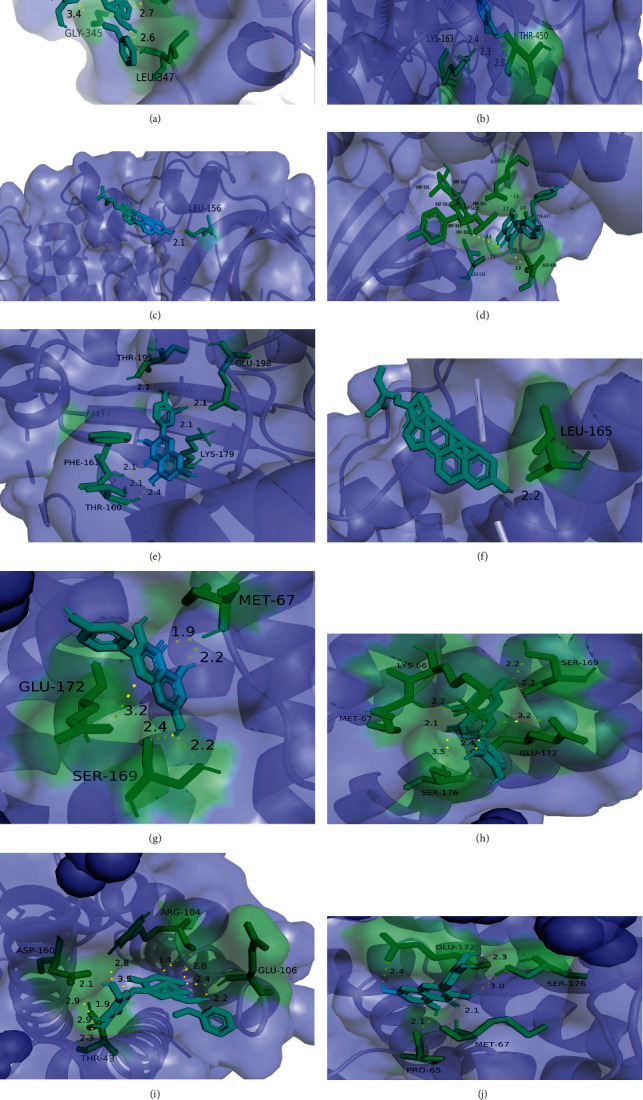
“Molecular docking 3D diagram. Small drug molecules are represented by sky blue sticks, hydrogen bonds by yellow dotted lines, and amino acid residues bound to small drug molecules by green sticks. (a) Paeoniflorin-AKT1 (binding energy = −8.35 kcal·mol-1). (b) Luteolin-AKT1 (binding energy = −8.61 kcal·mol-1). (c) Beta-sitosterol-AKT1 (binding energy = −10.09 kcal·mol-1). (d) Kaempferol-AKT1 (binding energy = −7.38 kcal·mol-1). (e) Quercetin-AKT1 (binding energy = −7.24 kcal·mol-1). (f) Beta-sitosterol-IL-6 (binding energy = −8.9 kcal·mol-1). (g) Kaempferol-IL-6 (binding energy = −8.17 kcal·mol-1). (h) Luteolin-IL-6 (binding energy = −7.34 kcal·mol-1). (i) Paeoniflorin-IL-6 (binding energy = −14.76 kcal·mol-1). (j) Quercetin-IL-6 (binding energy = −8.0 kcal·mol-1)”.

The strength of binding between hub genes and key active compounds was assessed by molecular docking. With binding energies less than −5 kJ/mol, the proteins in the hub genes are well connected to the key compounds. The 3D representation and binding energies of AKT1 and IL-6 docking are shown in Figure 7.

## 4. Discussion

Gou Qi Zi (Lycii Fructus), Fu Ling (Poria), Bai Shao (Paeoniae Radix Alba), Xian Ling Pi (Epimedii Folium), Huang Qi (Astragali Radix), Shu Di Huang (Rehmanniae Radix Praeparata), Gou Teng (Ramulus Uncariae cum Uncis), Muli (oysters), and He Huan Pi (Albizia Peel) are the nine TCM components of Yikunyin. According to Jae Hyun Kim et al., the water extract of Gou Qi Zi can inhibit RANKL-induced osteoclast differentiation and improve menopause osteoporosis [19]. Similarly, the combination of Rubus coreanus and Huang Qi can improve osteoporosis in ovariectomized mice [20]. Gou Teng alleviates menopause-induced depression in mice by activating the 5-HT1A receptor [21], and flavonoid-rich He Huan Pi suppresses oxidative stress in postmenopausal women [8, 22]. Finally, the Muli extract prevents bone loss due to ovariectomy [23]. Despite the established effects of different herbs, the mechanism of Yikunyin in treating menopausal syndrome remains unclear. In this study, the hub genes, key active compounds, and pathways implicated in the therapeutic activity of Yikunyin against menopausal syndrome are identified for the first time, using network pharmacology. The underlying mechanism of Yikunyin's therapeutic activity is also revealed.

The key active ingredients identified herein are paeoniflorin, luteolin, octadecatrienoic acid, beta-sitosterol, kaempferol, quercetin, acacic acid lactone, julibroside J24, julibrotriterpenoidal lactone A, norarmepavine, S-(2-carboxyethyl)-L-cysteine, and machaerinic acid lactone. Among these ingredients, paeoniflorin, luteolin, kaempferol, beta-sitosterol, and quercetin are noteworthy, as they can provide a reference for the future development of new drugs. Based on previous studies, luteolin inhibits insulin resistance, a condition caused by diseases (diabetes and metabolic syndrome) associated with the onset of menopause, by promoting the PI3K-Akt signaling pathway [24, 25]. Meanwhile, kaempferol reduces inflammation and lipid peroxidation, and thus, it may have a therapeutic effect on menopausal atherosclerosis [26, 27]. According to Yue-Hua Jiang et al., beta-sitosterol plays an important role in regulating anti-LDL and anti-atherosclerosis processes via PI3K-Akt signaling [28]. As regards paeoniflorin, it prevents atherosclerosis by inhibiting oxidized LDL [29] and alleviates atherosclerotic inflammation [30]. It also protects nerves by promoting Akt phosphorylation and exerting antidepression effects [31], and thus, it may have a positive therapeutic impact on menopausal depression. Finally, quercetin inhibits inflammation and slows atherosclerosis by regulating the PI3K-Akt signaling pathway [32]. To the best of our knowledge, no other active ingredients in Yikunyin have been reported in the literature. In conclusion, the effect of Yikunyin in treating menopausal syndrome is related to paeoniflorin, luteolin, kaempferol, beta-sitosterol, and quercetin key active ingredients.

The 15 hub genes identified herein are as follows: AKT1, PRKCA, TLR9, CXCL10, PRKCD, PARP1, ABCB1, TP53, CAV1, MAPK8, PPARA, GRB2, EGFR, IL-6, and JAK2. MCODE analysis shows that AKT1 and IL-6 played the most important roles. IL-6 was used to develop tocilizumab, a drug that clinically treats arthritis and alleviates the symptoms of COVID-19 by targeting interleukin-6 receptors [33]. According to Reeta Kangas and colleagues, the amount of AKT1 in the subcutaneous fat of postmenopausal women is lower than that in premenopausal women [34]. Considering that AKT1-deficient adipocytes are less sensitive to insulin [35], menopause may thus interfere with lipid storage, leading to lipid metabolism disorders. Studies conducted on beta-sitosterol show that it improves lipid metabolism in mice [36] and that it may antagonize oxidized LDL by targeting the AKT1 implicating PI3K-Akt signaling pathway [28]. In a study [37] involving 281 middle-aged (45–60 years old) healthy women, Huang et al. showed that poor sleep efficiency is associated with elevated levels of IL-6 and that menopausal animal models exhibit increased inflammatory response and elevated levels of IL-6 [38]. Paeoniflorin reduces IL-6 levels [39], and thus, it can improve sleep efficiency in menopausal women. Among the key active ingredients, quercetin and luteolin suppress CXCL10 expression at protein and mRNA levels [40], thereby inhibiting osteoclast differentiation and protecting against ovariectomy-induced bone loss [41], which is of great significance in the treatment of menopausal osteoporosis. The remaining core targets identified in our research have not yet been developed into drugs, and more research is needed to elucidate their therapeutic effects.

To further assess the complex relationships existing between different component, targets, and pathways implicated in the treatment of menopausal syndrome by Yikunyin, GO and KEGG analyses of the 15 key targets were conducted. The results of GO analysis show that the 15 targets are mainly enriched in phosphatase binding, cytokine receptor binding, cellular response to oxidative stress, and cellular response to chemical stress, with the former three biological processes being the most important. After entering menopause, the level of alkaline phosphatase in women increases significantly, and it keeps on increasing with time. This indicates that the bone turnover level, a marker of menopausal osteoporosis, also increases [42]. As mentioned above, Huang et al. report that menopausal animal models exhibit increased inflammatory responses and elevated levels of IL-6 [37, 38]. In general, high levels of IL-6 cytokine are associated with increased inflammatory reaction, which in turn is closely related to the occurrence and development of cancer [43]. Therefore, the regulation of IL-6 plays a key role in suppressing chronic inflammation in menopausal women, which ultimately reduces the incidence of cancer and slows down its progression. We speculate that the key active ingredients in Yikunyin (such as paeoniflorin) may inhibit inflammation in menopausal women by regulating cytokine (IL-6) receptor binding. In addition to inflammation, menopause is associated with lower estrogen levels. Therefore, menopause diminishes or eliminates the protective effects of estrogen against drug- or poison-induced damage. For example, estrogen protects the heart against the toxic effects of anthracyclines by regulating oxidative stress [44], an indicator that is also closely related to the development of cancer [45], and the advent of menopause inhibits this protective effect. Based on the available data, it may be hypothesized that Yikunyin can treat menopausal syndrome by regulating phosphatase binding, cytokine receptor binding, and oxidative stress responses.

The KEGG analysis results demonstrate that most of the 15 hub genes are involved in PI3K-Akt signaling, lipid and atherosclerosis, and hepatitis B, and the former seems to be the most critical pathway implicated in the therapeutic effect of Yikunyin on menopausal syndrome. Unlike Lee et al. [46], we believe that Yikunyin can alleviate the symptoms of menopause by upgrading the phosphorylation level of Akt. As mentioned above, a pronounced increase in alkaline phosphatase is observed in women after menopause [42]. Considering that phosphatase is a major antagonist of P13 K [47], the high levels of this enzyme in menopausal women may lead to excessive inhibition of the PI3K-Akt signaling pathway, resulting in a series of menopausal symptoms. A study conducted by Meng et al. suggests that the upregulation of PI3K-Akt signaling can reduce cholesterol accumulation, inhibit inflammatory signaling, and alleviate postmenopausal dyslipidemia [48]. Osteoarthritis, a disease occurring in women over the age of 50, often in menopause [4], may also be prevented upon the activation of the PI3K-Akt signaling pathway, which plays an important role in the development of postmenopausal osteoarthritis [49]. Based on previous reports, menopause is an additional risk factor for atherosclerosis [3]. Therefore, the prevention and improvement of atherosclerosis have a positive impact on the treatment of menopausal syndrome. Moreover, Lee et al. demonstrated that the phosphorylation of eNOS may be increased by regulating the PI3K-Akt signaling pathway, which ultimately results in the treatment of cardiovascular disease caused by vascular endothelial cell dysfunction [50]. The PI3K-Akt signaling pathway is also involved in the alleviation of depression, a condition that is more prevalent among postmenopausal women than among premenopausal ones [51, 52]. Luteolin and quercetin, two key active flavonoid compounds in Yikunyin, have antidepressant activity [53, 54], and thus, the natural Yikunyin prescription can potentially improve depression, one of the symptoms of menopausal syndrome. Previous studies that demonstrate the effect of menopause in increasing inflammation and IL-6 levels [38]suggest that the alleviation of excessive inflammatory response in menopausal women may have a positive effect on the treatment of menopausal atherosclerosis, a disease instigated by chronic inflammation [27]. They also highlight the importance of regulating cytokine levels, such as IL-6 levels. With structures similar to curcumin (phenolic O–H, unsaturated carbonyl, and C–H groups), flavonoids such as quercetin and luteolin are expected to have the same biological activity as curcumin, including antioxidant and anti-inflammatory activities [55]. Indeed, the anti-inflammation effect of these two compounds has been established in previous studies [56, 57]. Interestingly, the 15 hub genes identified herein are also significantly enriched in the hepatitis B pathway. Based on the available literature [58], the antiviral effect of hepatitis B in premenopausal women is better than that in postmenopausal women (*P* < 0.001), as manifested by the recovery of liver fibrosis. Moreover, curcumin, a compound whose structure is similar to those of quercetin and luteolin, has a hepatoprotective effect [55], and quercetin and kaempferol have been shown to inhibit the activity of the hepatitis B virus, which may interfere with hepatitis B virus proteins [59]. Therefore, we speculate that the key compounds in Yikunyin may have anti-hepatitis effects or can promote the antiviral treatment of hepatitis B, to eliminate the difference between the effects of this treatment before and after menopause. In summary, Yikunyin alleviates the symptoms of menopause mainly by regulating the PI3K-Akt signaling pathway, lipid and atherosclerosis, and hepatitis B.

## 5. Conclusion

This study highlights the importance of Yikunyin in improving menopausal syndrome by upregulating the PI3K-Akt signaling pathway, improving inflammatory response, promoting antioxidant, and adjusting lipid metabolism to prevent atherosclerosis. The most important active ingredients in Yikunyin are paeoniflorin, luteolin, kaempferol, beta-sitosterol, and quercetin. In addition, our research provides some new targets for the future development of new drugs.

## Data Availability

The data used to support the findings of this study are available from the corresponding author upon request.

## Supplementary Materials

Supplementary Materials Supplementary File 1: 121 potential active ingredients in Yikunyin screened by ADME. Supplementary File 2: molecular IDs corresponding to signs in circles. Supplementary File 3: ranking of 15 hub genes by 12 CytoHubba algorithms.
