# Supplementary material for: Network Pharmacology and Molecular Docking Analysis of the Mechanism Underlying Yikunyin's Therapeutic Effect on Menopausal Syndrome
Source: Evid Based Complement Alternat Med. 2022 Jun 6;2022:7302419. doi: 10.1155/2022/7302419 (PMC9192326; doi:10.1155/2022/7302419)
Supplement: Supplementary Materials — Supplementary File 1: 121 potential active ingredients in Yikunyin screened by ADME. Supplementary File 2: molecular IDs corresponding to signs in circles. Supplementary File 3: ranking of 15 hub genes by 12 CytoHubba algorithms. [file 7302419.f1.docx]

**Supplementary File 1: 121 potential active ingredients in Yikunyin screened by ADME**

**ID Molecule name OB(%) DL Source**

**MOL001910 11alpha,12alpha-epoxy-3beta- 64.77 0.38 Baishao**

**23-dihydroxy-30-norolean-20-**

**en-28,12beta-olide**

**MOL001918 paeoniflorgenone 87.59 0.37 Baishao**

**MOL001919 (3S,5R,8R,9R,10S,14S)-3,17- 43.56 0.53 Baishao**

**dihydroxy-4,4,8,10,14-**

**pentamethyl-2,3,5,6,7,9-**

**hexahydro-1H-**

**cyclopenta[a]phenanthrene-**

**15,16-dione**

**MOL001921 Lactiflorin 49.12 0.8 Baishao**

**MOL001924 paeoniflorin 53.87 0.79 Baishao**

**MOL001925 paeoniflorin_qt 68.18 0.4 Baishao**

**MOL001928 albiflorin_qt 66.64 0.33 Baishao**

**MOL001930 benzoyl paeoniflorin 31.27 0.75 Baishao**

**MOL000211 Mairin 55.38 0.78 Baishao**

**MOL000358 beta-sitosterol 36.91 0.75 Baishao**

**MOL000359 sitosterol 36.91 0.75 Baishao**

**MOL000422 kaempferol 41.88 0.24 Baishao**

**MOL000492 (+)-catechin 54.83 0.24 Baishao**

**MOL000358 beta-sitosterol 36.91 0.75 Gouteng**

**MOL000359 sitosterol 36.91 0.75 Gouteng**

**MOL000422 kaempferol 41.88 0.24 Gouteng**

**MOL000073 ent-Epicatechin 48.96 0.24 Gouteng**

**MOL008455 3-oxo-22伪-hydroxyurs-12-en-**

**27,28-dioc acid 32.33 0.68 Gouteng**

**MOL008456 (3E,4R)-4-(1,3-benzodioxol-5-**

**ylmethyl)-3-[(3,4,5-**

**trimethoxyphenyl)methylidene]**

**oxolan-2-one 51.78 0.65 Gouteng**

**MOL008457 Tetrahydroalstonine 32.42 0.81 Gouteng**

**MOL008458 Angustidine 51.85 0.66 Gouteng**

**MOL008460 geissoschizinc acid 49.92 0.6 Gouteng**

**MOL008463 SMR000232338 56.74 0.75 Gouteng**

**MOL008465 (E)-16,17-Didehydro-17-methoxy**

**-17,18-seco-3-beta-yohimban-16**

**-carboxylic acid methyl ester 32.75 0.64 Gouteng**

**MOL008467 Rhynchophylline A 68.68 0.69 Gouteng**

**MOL008468 methyl(E)-2-[(2S,3Z,12bS)-3-**

**ethylidene-2,4,6,7,12,12b-**

**hexahydro-1H-indolo[3,2**

**-h]quinolizin-2-yl]-3-methoxyprop**

**-2-enoate 56.83 0.64 Gouteng**

**MOL008469 Rhynchophylline 41.82 0.57 Gouteng**

**MOL008470 SMR000232333 78.38 0.75 Gouteng**

**MOL008471 Isorhyncophylline 47.31 0.57 Gouteng**

**MOL008472 hirsutasideA 70.34 0.81 Gouteng**

**MOL008473 (E)-2-[(3S,6'S,7'S,8'aS)-6'-ethyl-**

**2-keto-spiro[indoline-3,1'-**

**indolizidine]-7'-yl]-3-methoxy-**

**acrylic acid methyl ester 57.85 0.57 Gouteng**

**MOL008474 (E)-2-[(3R,6'S,7'S,8'aS)-6'-ethyl-**

**2-keto-spiro[indoline-3,1'-**

**indolizidine]-7'-yl]-3-methoxy-**

**acrylic acid methyl ester 54.47 0.57 Gouteng**

**MOL008475 Mitraphyllic acid 31.7 0.7 Gouteng**

**MOL008476 hirsutasideB 40.21 0.8 Gouteng**

**MOL008477 corynoxeine 57.13 0.57 Gouteng**

**MOL008478 methyl(E)-2-[(2S,3R,12bS)-**

**3-vinyl-1,2,3,4,6,7,12,12b-**

**octahydroindolo[3,2-h]quinolizin-**

**2-yl]-3-methoxy-prop-2-enoate 31.94 0.64 Gouteng**

**MOL008481 (1'R,3S,4a'S,5a'S,10a'R)-1'-methyl-**

**2-oxo-1',4a',5',5a',7',8',10',10a'-**

**octahydrospiro[indoline-3,6'-**

**pyrano[3,4-f]indolizine]-4'-**

**carboxylic acid 105.22 0.7 Gouteng**

**MOL008482 (2S,12bR)-methyl2-((E)-1-oxobut**

**-2-en-2-yl)-1,2,6,7,12,12b-**

**hexahydroindolo[2,3-a]quinolizine-**

**3-carboxylate 42.07 0.6 Gouteng**

**MOL008484 vincoside lactam_qt 50.81 0.82 Gouteng**

**MOL008485 hirsutasideC 34.27 0.75 Gouteng**

**MOL008487 hirsutine 34.44 0.43 Gouteng**

**MOL008488 yohimbine 46.42 0.81 Gouteng**

**MOL008489 delta(sup 18)-Hirsutine 41.64 0.64 Gouteng**

**MOL008490 isocorynantheic acid 72.36 0.6 Gouteng**

**MOL000098 quercetin 46.43 0.28 Gouteng**

**MOL008635 coryincine 38.27 0.81 Gouteng**

**MOL001323 Sitosterol alpha1 43.28 0.78 Gouyqizi**

**MOL003578 Cycloartenol 38.69 0.78 Gouyqizi**

**MOL001494 Mandenol 42 0.19 Gouyqizi**

**MOL001495 Ethyl linolenate 46.1 0.2 Gouyqizi**

**MOL001979 LAN 42.12 0.75 Gouyqizi**

**MOL000449 Stigmasterol 43.83 0.76 Gouyqizi**

**MOL000358 beta-sitosterol 36.91 0.75 Gouyqizi**

**MOL005406 atropine 45.97 0.19 Gouyqizi**

**MOL005438 campesterol 37.58 0.71 Gouyqizi**

**MOL006209 cyanin 47.42 0.76 Gouyqizi**

**MOL007449 24-methylidenelophenol 44.19 0.75 Gouyqizi**

**MOL008173 daucosterol_qt 36.91 0.75 Gouyqizi**

**MOL008400 glycitein 50.48 0.24 Gouyqizi**

**MOL010234 delta-Carotene 31.8 0.55 Gouyqizi**

**MOL000953 CLR 37.87 0.68 Gouyqizi**

**MOL009604 14b-pregnane 34.78 0.34 Gouyqizi**

**MOL009612 (24R)-4alpha-Methyl-24-**

**ethylcholesta-7,25-dien-3beta-**

**ylacetate 46.36 0.84 Gouyqizi**

**MOL009615 24-Methylenecycloartan-**

**3beta,21-diol 37.32 0.8 Gouyqizi**

**MOL009617 24-ethylcholest-22-enol 37.09 0.75 Gouyqizi**

**MOL009618 24-ethylcholesta-5,22-dienol 43.83 0.76 Gouyqizi**

**MOL009620 24-methyl-31-norlanost-9(11)-**

**enol 38 0.75 Gouyqizi**

**MOL009621 24-methylenelanost-8-enol 42.37 0.77 Gouyqizi**

**MOL009622 Fucosterol 43.78 0.76 Gouyqizi**

**MOL009631 31-Norcyclolaudenol 38.68 0.81 Gouyqizi**

**MOL009633 31-norlanost-9(11)-enol 38.35 0.72 Gouyqizi**

**MOL009634 31-norlanosterol 42.2 0.73 Gouyqizi**

**MOL009635 4,24-methyllophenol 37.83 0.75 Gouyqizi**

**MOL009639 Lophenol 38.13 0.71 Gouyqizi**

**MOL009640 4alpha,14alpha,24-trimethylcholesta**

**-8,24-dienol 38.91 0.76 Gouyqizi**

**MOL009641 4alpha,24-dimethylcholesta-7,24**

**-dienol 42.65 0.75 Gouyqizi**

**MOL009642 4alpha-methyl-24-ethylcholesta**

**-7,24-dienol 42.3 0.78 Gouyqizi**

**MOL009644 6-Fluoroindole-7-**

**Dehydrocholesterol 43.73 0.72 Gouyqizi**

**MOL009646 7-O-Methylluteolin-6-C-beta-**

**glucoside_qt 40.77 0.3 Gouyqizi**

**MOL009650 Atropine 42.16 0.19 Gouyqizi**

**MOL009651 Cryptoxanthin monoepoxide 46.95 0.56 Gouyqizi**

**MOL009653 Cycloeucalenol 39.73 0.79 Gouyqizi**

**MOL009656 (E,E)-1-ethyl octadeca-3,13-**

**dienoate 42 0.19 Gouyqizi**

**MOL009660 methyl(1R,4aS,7R,7aS)-4a,7-**

**dihydroxy-7-methyl-1-**

**[(2S,3R,4S,5S,6R)-3,4,5-trihydroxy**

**-6-(hydroxymethyl)oxan-2-yl]oxy-**

**1,5,6,7a-tetrahydrocyclopenta[d]pyran**

**-4-carboxylate 39.43 0.47 Gouyqizi**

**MOL009662 Lantadene A 38.68 0.57 Gouyqizi**

**MOL009664 Physalin A 91.71 0.27 Gouyqizi**

**MOL009665 Physcion-8-O-beta-D-**

**gentiobioside 43.9 0.62 Gouyqizi**

**MOL009677 lanost-8-en-3beta-ol 34.23 0.74 Gouyqizi**

**MOL009678 lanost-8-enol 34.23 0.74 Gouyqizi**

**MOL009681 Obtusifoliol 42.55 0.76 Gouyqizi**

**MOL000098 quercetin 46.43 0.28 Gouyqizi**

**MOL000359 sitosterol 36.91 0.75 Shudi**

**MOL000449 Stigmasterol 43.83 0.76 Shudi**

**MOL000273 (2R)-2-[(3S,5R,10S,13R,14R,16R,**

**17R)-3,16-dihydroxy-4,4,10,13,14**

**-pentamethyl-2,3,5,6,12,15,16,17**

**-octahydro-1H-cyclopenta[a]**

**phenanthren-17-yl]-6-methylhept**

**-5-enoic acid 30.93 0.81 Fuling**

**MOL000275 trametenolic acid 38.71 0.8 Fuling**

**MOL000276 7,9(11)-dehydropachymic acid 35.11 0.81 Fuling**

**MOL000279 Cerevisterol 37.96 0.77 Fuling**

**MOL000280 (2R)-2-[(3S,5R,10S,13R,14R,16R,**

**17R)-3,16-dihydroxy-4,4,10,13,14**

**-pentamethyl-2,3,5,6,12,15,16,17**

**-octahydro-1H-cyclopenta[a]**

**phenanthren-17-yl]-5-isopropyl-**

**hex-5-enoic acid 31.07 0.82 Fuling**

**MOL000282 ergosta-7,22E-dien-3beta-ol 43.51 0.72 Fuling**

**MOL000283 Ergosterol peroxide 40.36 0.81 Fuling**

**MOL000285 (2R)-2-[(5R,10S,13R,14R,16R,17R)**

**-16-hydroxy-3-keto-4,4,10,13,14**

**-pentamethyl-1,2,5,6,12,15,16,17-octahydrocyclopenta[a]phenanthren-**

**17-yl]-5-isopropyl-hex-**

**5-enoic acid 38.26 0.82 Fuling**

**MOL000287 3beta-Hydroxy-24-methylene-8-**

**lanostene-21-oic acid 38.7 0.81 Fuling**

**MOL000289 pachymic acid 33.63 0.81 Fuling**

**MOL000290 Poricoic acid A 30.61 0.76 Fuling**

**MOL000291 Poricoic acid B 30.52 0.75 Fuling**

**MOL000292 poricoic acid C 38.15 0.75 Fuling**

**MOL000296 hederagenin 36.91 0.75 Fuling**

**MOL000300 dehydroeburicoic acid 44.17 0.83 Fuling**

**MOL000211 Mairin 55.38 0.78 Huangqi**

**MOL000239 Jaranol 50.83 0.29 Huangqi**

**MOL000296 hederagenin 36.91 0.75 Huangqi**

**MOL000033 (3S,8S,9S,10R,13R,14S,17R)-10,13-**

**dimethyl-17-[(2R,5S)-5-propan-2-**

**yloctan-2-yl]-2,3,4,7,8,9,11,12,14,15,**

**16,17-dodecahydro-1H-cyclopenta[a]**

**phenanthren-3-ol 36.23 0.78 Huangqi**

**MOL000354 isorhamnetin 49.6 0.31 Huangqi**

**MOL000371 3,9-di-O-methylnissolin 53.74 0.48 Huangqi**

**MOL000374 5'-hydroxyiso-muronulatol-2',5'-di-**

**O-glucoside 41.72 0.69 Huangqi**

**MOL000378 7-O-methylisomucronulatol 74.69 0.3 Huangqi**

**MOL000379 9,10-dimethoxypterocarpan-3-O-尾**

**-D-glucoside 36.74 0.92 Huangqi**

**MOL000380 (6aR,11aR)-9,10-dimethoxy-6a,11a-**

**dihydro-6H-benzofurano[3,2-c]chromen**

**-3-ol 64.26 0.42 Huangqi**

**MOL000387 Bifendate 31.1 0.67 Huangqi**

**MOL000392 formononetin 69.67 0.21 Huangqi**

**MOL000398 isoflavanone 109.99 0.3 Huangqi**

**MOL000417 Calycosin 47.75 0.24 Huangqi**

**MOL000422 kaempferol 41.88 0.24 Huangqi**

**MOL000433 FA 68.96 0.71 Huangqi**

**MOL000438 (3R)-3-(2-hydroxy-3,4-dimethoxyphenyl)**

**chroman-7-ol 67.67 0.26 Huangqi**

**MOL000439 isomucronulatol-7,2'-di-O-glucosiole 49.28 0.62 Huangqi**

**MOL000442 1,7-Dihydroxy-3,9-dimethoxy**

**pterocarpene 39.05 0.48 Huangqi**

**MOL000098 quercetin 46.43 0.28 Huangqi**

**MOL001510 24-epicampesterol 37.58 0.71 Xianlingpi**

**MOL001645 Linoleyl acetate 42.1 0.2 Xianlingpi**

**MOL001771 poriferast-5-en-3beta-ol 36.91 0.75 Xianlingpi**

**MOL001792 DFV 32.76 0.18 Xianlingpi**

**MOL003044 Chryseriol 35.85 0.27 Xianlingpi**

**MOL003542 8-Isopentenyl-kaempferol 38.04 0.39 Xianlingpi**

**MOL000359 sitosterol 36.91 0.75 Xianlingpi**

**MOL000422 kaempferol 41.88 0.24 Xianlingpi**

**MOL004367 olivil 62.23 0.41 Xianlingpi**

**MOL004373 Anhydroicaritin 45.41 0.44 Xianlingpi**

**MOL004380 C-Homoerythrinan,1,6-didehydro-**

**3,15,16-trimethoxy-, (3.beta.)- 39.14 0.49 Xianlingpi**

**MOL004382 Yinyanghuo A 56.96 0.77 Xianlingpi**

**MOL004384 Yinyanghuo C 45.67 0.5 Xianlingpi**

**MOL004386 Yinyanghuo E 51.63 0.55 Xianlingpi**

**MOL004388 6-hydroxy-11,12-dimethoxy-2,2-**

**dimethyl-1,8-dioxo-2,3,4,8-tetrahydro**

**-1H-isochromeno[3,4-h]isoquinolin-2**

**-ium 60.64 0.66 Xianlingpi**

**MOL004391 8-(3-methylbut-2-enyl)-2-phenyl**

**-chromone 48.54 0.25 Xianlingpi**

**MOL004394 Anhydroicaritin-3-O-alpha-L**

**-rhamnoside 41.58 0.61 Xianlingpi**

**MOL004396 1,2-bis(4-hydroxy-3**

**-methoxyphenyl)propan-1,3**

**-diol 52.31 0.22 Xianlingpi**

**MOL004425 Icariin 41.58 0.61 Xianlingpi**

**MOL004427 Icariside A7 31.91 0.86 Xianlingpi**

**MOL000006 luteolin 36.16 0.25 Xianlingpi**

**MOL000622 Magnograndiolide 63.71 0.19 Xianlingpi**

**MOL000098 quercetin 46.43 0.28 Xianlingpi**

**HBIN014312 acacicacid lactone** **/ / Hehuanpi**

**HBIN031622 julibroside j24 / / Hehuanpi**

**HBIN031632 julibrotriterpenoidal lactone a / / Hehuanpi**

**HBIN037292 norarmepavine / / Hehuanpi**

**HBIN042696 s-(2-carboxyethyl)-l-cysteine / / Hehuanpi**

**HBIN034090 machaerinic acid lactone / / Hehuanpi**

[**HBIN034091**](http://herb.ac.cn/Detail/?v=HBIN034091&label=Ingredient) **machaerinic acid methyl ester / / Hehuanpi**

**HBIN034100 macluraxanthone / / Hehuanpi**

**HBIN048047 vitamin c / / Hehuanpi**

**HBIN037285 noradrenaline / / Hehuanpi**

**/ cysteine** **/ / Muli**

**/ docosahexenoic acid / / Muli**

**/ glutamic acid / / Muli**

**/ octadecatrienoic acid / / Muli**

**/ asparagic acid / / Muli**

**/ vitamin A / / Muli**

**/ fucose / / Muli**

**Supplementary File 2: molecular IDs corresponding to signs in circles.**

| **Sign** | **Molecular ID** |
| --- | --- |
| **BS1** | **MOL000492** |
| **BS2** | **MOL001919** |
| **BS3** | **MOL001924** |
| **FL1** | **MOL000273** |
| **FL2** | **MOL000275** |
| **FL3** | **MOL000279** |
| **FL4** | **MOL000282** |
| **FL5** | **MOL000283** |
| **GT1** | **MOL000073** |
| **GT2** | **MOL008456** |
| **GT3** | **MOL008457** |
| **GT4** | **MOL008458** |
| **GT5** | **MOL008460** |
| **GT6** | **MOL008463** |
| **GT7** | **MOL008465** |
| **GT8** | **MOL008467** |
| **GT9** | **MOL008468** |
| **GT10** | **MOL008469** |
| **GT11** | **MOL008470** |
| **GT12** | **MOL008471** |
| **GT13** | **MOL008472** |
| **GT14** | **MOL008473** |
| **GT15** | **MOL008474** |
| **GT16** | **MOL008475** |
| **GT17** | **MOL008476** |
| **GT18** | **MOL008477** |
| **GT19** | **MOL008478** |
| **GT20** | **MOL008481** |
| **GT21** | **MOL008482** |
| **GT22** | **MOL008484** |
| **GT23** | **MOL008485** |
| **GT24** | **MOL008487** |
| **GT25** | **MOL008488** |
| **GT26** | **MOL008489** |
| **GT27** | **MOL008490** |
| **GT28** | **MOL008635** |
| **GQZ1** | **MOL000953** |
| **GQZ2** | **MOL001323** |
| **GQZ3** | **MOL001494** |
| **GQZ4** | **MOL001495** |
| **GQZ5** | **MOL001979** |
| **GQZ6** | **MOL003578** |
| **GQZ7** | **MOL005406** |
| **GQZ8** | **MOL005438** |
| **GQZ9** | **MOL006209** |
| **GQZ10** | **MOL007449** |
| **GQZ11** | **MOL008173** |
| **GQZ12** | **MOL008400** |
| **GQZ13** | **MOL009604** |
| **GQZ14** | **MOL009617** |
| **GQZ15** | **MOL009618** |
| **GQZ16** | **MOL009620** |
| **GQZ17** | **MOL009621** |
| **GQZ18** | **MOL009622** |
| **GQZ19** | **MOL009633** |
| **GQZ20** | **MOL009634** |
| **GQZ21** | **MOL009635** |
| **GQZ22** | **MOL009639** |
| **GQZ23** | **MOL009640** |
| **GQZ24** | **MOL009641** |
| **GQZ25** | **MOL009642** |
| **GQZ26** | **MOL009644** |
| **GQZ27** | **MOL009646** |
| **GQZ28** | **MOL009650** |
| **GQZ29** | **MOL009656** |
| **GQZ30** | **MOL009677** |
| **GQZ31** | **MOL009678** |
| **GQZ32** | **MOL009681** |
| **HQ1** | **MOL000033** |
| **HQ2** | **MOL000239** |
| **HQ3** | **MOL000354** |
| **HQ4** | **MOL000371** |
| **HQ5** | **MOL000378** |
| **HQ6** | **MOL000379** |
| **HQ7** | **MOL000380** |
| **HQ8** | **MOL000387** |
| **HQ9** | **MOL000392** |
| **HQ10** | **MOL000417** |
| **HQ11** | **MOL000433** |
| **HQ12** | **MOL000442** |
| **XLP1** | **MOL000006** |
| **XLP2** | **MOL001510** |
| **XLP3** | **MOL001645** |
| **XLP4** | **MOL001771** |
| **XLP5** | **MOL001792** |
| **XLP6** | **MOL003044** |
| **XLP7** | **MOL003542** |
| **XLP8** | **MOL004367** |
| **XLP9** | **MOL004373** |
| **XLP10** | **MOL004380** |
| **XLP11** | **MOL004382** |
| **XLP12** | **MOL004384** |
| **XLP13** | **MOL004386** |
| **XLP14** | **MOL004388** |
| **XLP15** | **MOL004391** |
| **XLP16** | **MOL004396** |
| **XLP17** | **MOL004427** |
| **ML1** | **cysteine** |
| **ML2** | **docosahexenoic acid** |
| **ML3** | **glutamic acid** |
| **ML4** | **octadecatrienoic acid** |
| **ML5** | **asparagic acid** |
| **ML6** | **vitamin A** |
| **ML7** | **fucose** |
| **HHP1** | **HBIN014312** |
| **HHP2** | **HBIN031622** |
| **HHP3** | **HBIN031632** |
| **HHP4** | **HBIN034090** |
| **HHP5** | **HBIN034091** |
| **HHP6** | **HBIN034100** |
| **HHP7** | **HBIN037285** |
| **HHP8** | **HBIN037292** |
| **HHP9** | **HBIN042696** |
| **HHP10** | **HBIN048047** |
| **A1** | **MOL000422** |
| **A2** | **MOL000358** |
| **A3** | **MOL000359** |
| **A4** | **MOL000211** |
| **B** | **MOL000296** |
| **C** | **MOL000098** |
| **D** | **MOL000449** |

**Supplementary File 3: The ranking of 15 hub genes by 12 CytiHubba algorithms.**

**Top 10 in network string_interactions%20(7).tsv ranked by Betweenness method**

Rank Name Score

1 AKT1 3563.216202

2 PRKCA 3533.37596

3 TP53 3039.641081

4 EGFR 2887.647333

5 HSP90AA1 2657.297788

6 JAK2 2601.742892

7 AR 2460.976835

8 JUN 2328.74713

9 ESR1 2231.896419

10 IL6 2108.399322

**Top 10 in network string_interactions%20(7).tsv ranked by BottleNeck method**

Rank Name Score

1 PRKCA 15

2 TNF 12

3 ESR1 10

4 AKT1 9

4 JUN 9

4 IL6 9

7 VEGFA 8

8 EGFR 7

8 JAK2 7

8 AR 7

**Top 10 in network string_interactions%20(7).tsv ranked by Closeness method**

Rank Name Score

1 AKT1 143.0833333

2 TP53 142.1666667

3 JUN 138.75

4 HSP90AA1 138

5 EGFR 136.75

6 TNF 135.5

7 MAPK1 133.9166667

8 IL6 133.2833333

9 RELA 131.9166667

10 ESR1 130.5833333

**Top 10 in network string_interactions%20(7).tsv ranked by ClusteringCoefficient method**

Rank Name Score

1 TLR9 0.454545455

2 PPARD 0.428571429

2 CHRNA2 0.428571429

4 KDM1A 0.418300654

5 E2F2 0.417582418

6 ACACA 0.4

6 AKR1C2 0.4

6 ALOX12 0.4

6 PLA2G2A 0.4

6 CHRNB4 0.4

**Top 10 in network string_interactions%20(7).tsv ranked by Degree method**

Rank Name Score

1 AKT1 146

2 TP53 140

3 JUN 122

4 HSP90AA1 120

5 TNF 118

6 EGFR 114

7 IL6 112

8 MAPK1 104

9 RELA 98

10 CASP3 96

**Top 10 in network string_interactions%20(7).tsv ranked by DMNC method**

Rank Name Score

1 CXCL10 1.579460238

2 IL1A 1.567959121

3 KDM1A 1.52745248

4 IKBKB 1.507225761

5 IL2 1.456324857

6 VDR 1.426474883

6 TLR9 1.426474883

8 MMP1 1.41890358

9 PTGS2 1.41398737

10 JAK3 1.408594804

**Top 10 in network string_interactions%20(7).tsv ranked by EcCentricity method**

Rank Name Score

1 PRKCA 0.24787234

1 PRKCD 0.24787234

1 PARP1 0.24787234

1 ABCB1 0.24787234

1 TP53 0.24787234

1 CAV1 0.24787234

1 MAPK8 0.24787234

1 PPARA 0.24787234

1 GRB2 0.24787234

1 EGFR 0.24787234

**Top 10 in network string_interactions%20(7).tsv ranked by EPC method**

Rank Name Score

1 AKT1 64.103

2 JUN 63.83

3 TP53 63.438

4 HSP90AA1 61.98

5 RELA 61.735

6 TNF 61.477

7 IL6 61.049

8 EGFR 60.678

9 MYC 59.587

10 MAPK1 58.662

**Top 10 in network string_interactions%20(7).tsv ranked by MCC method**

Rank Name Score

1 IL6 9.75E+07

2 TNF 9.68E+07

3 IL1B 9.47E+07

4 CXCL8 9.39E+07

5 CCL2 9.32E+07

6 IL4 9.15E+07

7 ICAM1 8.86E+07

8 IL2 8.85E+07

9 IL1A 8.79E+07

10 IL10 8.59E+07

**Top 10 in network string_interactions%20(7).tsv ranked by MNC method**

Rank Name Score

1 AKT1 71

2 TP53 70

3 JUN 61

4 HSP90AA1 59

4 TNF 59

6 EGFR 56

7 IL6 55

8 MAPK1 52

9 RELA 49

10 CASP3 48

**Top 10 in network string_interactions%20(7).tsv ranked by Radiality method**

Rank Name Score

1 TP53 6.034409391

2 AKT1 6.03013573

3 HSP90AA1 6.025862069

3 JUN 6.025862069

5 EGFR 6.021588408

6 MAPK1 5.991672781

7 RELA 5.961757153

8 TNF 5.957483492

9 ESR1 5.931841526

10 IL6 5.919020543

**Top 10 in network string_interactions%20(7).tsv ranked by Stress method**

Rank Name Score

1 JAK2 787920

2 PRKCA 597496

3 AR 538272

4 AKT1 432128

5 EGFR 409496

6 TP53 397712

7 ESR1 379128

8 IL6 376616

9 JUN 362576

10 HSP90AA1 352280
